# Quality of life and persisting symptoms in intensive care unit survivors: implications for care after discharge

**DOI:** 10.1186/1756-0500-2-160

**Published:** 2009-08-12

**Authors:** Fiona J Baldwin, Denise Hinge, Joanna Dorsett, Owen F Boyd

**Affiliations:** 1 Intensive Care Unit, Royal Sussex County Hospital, Eastern Road, Brighton, UK

## Abstract

**Background:**

We assessed the quality of life of ICU survivors using SF-36 at 4 months after ICU discharge and investigated any correlation of PCS and MCS with age, illness severity and hospital or ICU length of stay. We examined the relationship between these variables, persisting physical and psychological symptoms and the perceived benefit of individual patients of follow-up.

**Findings:**

For one year, adult patients admitted for multiple organ or advanced respiratory support for greater than 48 hours to a 16-bedded teaching hospital general intensive care unit were identified. Those surviving to discharge were sent a questionnaire at 4 months following ICU discharge assessing quality of life and persisting symptoms. Demographic, length of stay and illness severity data were recorded. Higher or lower scores were divided at the median value. A two-tailed Students t-test assuming equal variances was used for normally-distributed data and Mann-Whitney tests for non-parametric data.

87 of 175 questionnaires were returned (50%), but only 65 had sufficient data giving a final response rate of 37%. Elderly patients had increased MCS as compared with younger patients. The PCS was inversely related to hospital LOS. There was a significant correlation between the presence of psychological and physical symptoms and desire for follow-up.

**Conclusion:**

Younger age and prolonged hospital stay are associated with lower mental or physical quality of life and may be targets for rehabilitation. Patients with persisting symptoms at 4 months view follow-up as beneficial and a simple screening questionnaire may identify those likely to attend outpatient services.

## Background

It is increasingly recognised that intensive care unit (ICU) survivors may have a reduction in quality of life [1,2].

Follow-up of survivors of ICU treatment has shown many patients suffer long-term physical and psychological consequences [3-5], although the prevalence of these symptoms and the role of rehabilitation services remains unclear.

Health-related quality of life (HRQOL) is a state of physical, mental and social wellbeing which is used in assessment of the longer term consequences of treatment [1]. There is no HRQOL score specific to the ICU population but a number of general scores have been evaluated [6,7]. Studies assessing HRQOL after intensive care have suggested it is reduced both in comparison to before admission [8-10] and in comparison to the general population [11,12], although it improves with time [8,10].

Studies assessing factors predicting a lower HRQOL following ICU admission, have generated more mixed results [6], which may represent the heterogeneity of both study groups and assessment tools used in ICU outcome research.

We assessed the quality of life of general ICU survivors using the Short Form 36 (SF-36) at 4 months post ICU discharge and investigated any correlation with age, illness severity and hospital or ICU length of stay. We wished to investigate any predictors of low HRQOL scores and identify patients who would potentially benefit from a follow up clinic.

SF-36 is a generic QOL instrument so we supplemented the questionnaire with further enquiries about persisting physical and psychological symptoms which were generated by a combination of literature review and local interest [5,13]. Aware of the high nonattendance rates at ICU clinics [14], we also asked patients to rate the perceived usefulness to them of the introduction of a follow-up service at our institution and investigated any relationship with persisting symptoms or SF 36-derived variables.

## Methods

The study was approved by Brighton and Mid Sussex Local Research Ethics Committee (MS 03/28). From November 2004 to October 2005 all adult patients admitted for multiple organ or advanced respiratory support for a period of greater than 48 hours to a 16-bedded university teaching hospital general intensive care unit were identified.

Patients surviving to ICU discharge were approached for verbal consent to participate in the study by a member of the Critical Care team and presented with an information sheet describing the study. We excluded all patients from outside the local area for whom we were unable to check for death after hospital discharge in order to avoid distress to next of kin and patients transferred from or to other ICUs.

We recorded standard demographic data for all ICU patients admitted for the period as well as illness severity (APACHE II score) [15], ICU length of stay (LOS), hospital LOS, mortality status and readmissions to ICU or hospital. Readmission was defined as a return to either hospital or the ICU having previously been discharged at any time during the four month period of the study. This was to identify patients less able to participate in the study. ICU and hospital LOS were calculated as days spent either on ICU or in an acute hospital. ICU LOS was not included in hospital LOS.

We posted a questionnaire to all participants four months after ICU discharge. The questionnaire incorporated both the SF36 (with permission of QualityMetric Incorporated, Lincoln, USA) and questions about persisting symptoms and desire for a local follow-up clinic. Patients were asked "Have you suffered from any of the following in the previous month?" The symptom list consisted of swallowing difficulties; loss of hearing; distressing memories popping into your mind; sleep difficulties; poor concentration; mood changes; weight loss. The patients were given no details about potential follow-up services.

There is consensus that the SF-36 is one of two instruments suited for measuring quality of life after critical care [1]. The SF-36 yields an 8-scale profile of functional health and well-being scores as well as psychometrically-based physical and mental health summary measures [16].

The SF-36 has demonstrated reliability and validity the critically ill [12,17] and the physical (PCS) and mental component summary (MCS) scores relating to quality of life have been used to summarise findings in longitudinal studies of mixed population ICU survivors [5,8,18].

The patients were provided with a stamped, self-addressed envelope in which to return the questionnaire. The questionnaire was piloted on 22 patients and these patients were not included in the final analysis.

Calculation of the SF-36 PCS and MCS scores were in accordance with standard techniques and using QualityMetric software to transform results to "norm based data". This adapts the data to show 50 as population mean and 10 representing one standard deviation [19].

### Statistical analysis

Results from the SF-36 were compared between the patients with the higher scores and those with the lower scores for the discriminator: age, APACHE II score, length of ICU stay (days), and hospital LOS (days). Higher or lower scores were divided at the median value. A two sample, two-tailed Students t-test assuming equal variances was used, p < 0.05 was considered significant (*). Results were compared between the lower 50% and higher 50% of patients for each discriminator.

The perceived benefit of follow up services was rated according to a five point scale (Table 1) and groups with or without persisting symptoms compared using the Mann-Whitney test converted to 2-tailed p value.

## Results

363 patients required multiple organ or advanced respiratory support with length of stay > 48 hours during the study period and of these 219 survived to 4 months following ICU discharge. 175 had returned home 4 months after ICU discharge. 87 questionnaires were returned with some data giving a response rate of 50%. The entire 175 were analyzed to explore the differences between the patients who responded to the questionnaire as compared to the non-responders (Table 2). Only 65 questionnaires had sufficient data to allow calculation of MCS and PCS scores which gives a response rate of 37% for summary score data (Table 3). Elderly patients had increased mental component summary as compared with younger patients. The physical component summary was inversely related to hospital LOS.

**Table 1:** Responses to: "How beneficial do you think a follow-up service would have been to you?"

**Response**	**Score rating**	**All patients** **n = 83**
Not at all	1	8

A little	2	13

Moderately	3	14

Quite a bit	4	23

Extremely	5	25

83 out of 87 patients responded to this question.

**Table 2:** Differences between patients responding and not responding to the questionnaire sent to them

	**All Responders**	**Full data Responders**	**All Non-responders**
Number	87	65	88

Age (median years)	66 (55–73)	63 (53–73)	59 (41–71)

Sex	32% Female	29% Female	34% Female

APACHE II (median)	18 (15–24)	17 (14–22)	17 (12–20)

ICU length of stay (median days)	8 (3–16)	8 (3–14)	5 (3–12)

Hospital length of stay (median days)	25 (15–48)	25 (14–47)	24 (14–36)

Hospital readmission rate	24%	26%	34%

ICU readmission rate	3.5%	4.6%	10%

Characteristics of both groups of responders described; all responders including those with partial data and full responders defined as those with sufficient data to allow PCS and MCS to be calculated. Interquartile range in brackets.

**Table 3:** MCS and PCS relationship to discriminators of age, length of stay and illness severity.

	Discriminator	Age (years)	Hospital length of stay (days)	ICU length of stay (days)	APACHE II
MCS	Mean below median value	39.32* (14.3)	44.99 (14.7)	42.12 (15.2)	44.04 (16.2)
	
	Mean above median value	**46.78 (14.7)**	41.28 (15.1)	44.07 (14.8)	42.21 (13.7)

PCS	Mean below median value	35.04 (11.7)	39.31* (12.8)	36.08 (11.9)	35.85 (12.1)
	
	Mean above median value	36.36 (11.5)	**32.22 (9.0)**	35.35 (11.4)	35.60 (11.1)

Relationship defined by the median of each variable in the responder patients (see Table 2). Standard deviations given in brackets. Patients above median age show improved SF-36 mental component scores. Patients above hospital median LOS have lower physical component scores. (* indicates statistical significance 2-tailed Student's t-test < 0.05. See text)

The desire for a follow-up service was rated on a 5-point scoring system and was completed by 83 patients (47%). There was no correlation between the perceived benefit of follow up services and demographic variables, illness severity or LOS, PCS, MCS or PCS and MCS combined.

**Figure 1:**
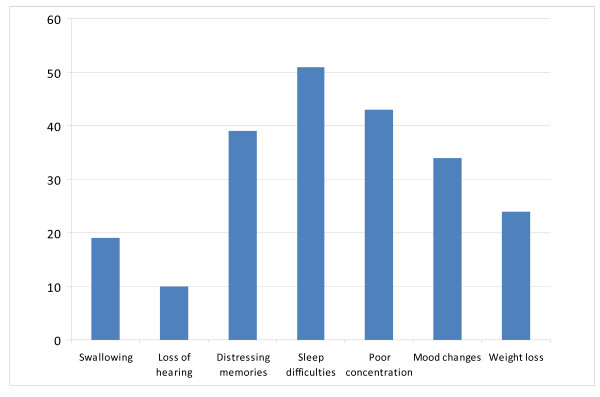
**Persisting symptoms related to ICU admission at 4 months**. The number of patients having persisting symptoms as described in Methods in the previous 1 month. Many patients had more than one symptom.

The persistence of symptoms in our population is illustrated in Figure 1. These symptoms were divided into psychological (distressing memories, sleep difficulties, poor concentration and mood changes) and physical groupings (dysphagia, weight loss and loss of hearing) in an attempt to mirror the PCS and MCS broad categorizations.

**Figure 2:**
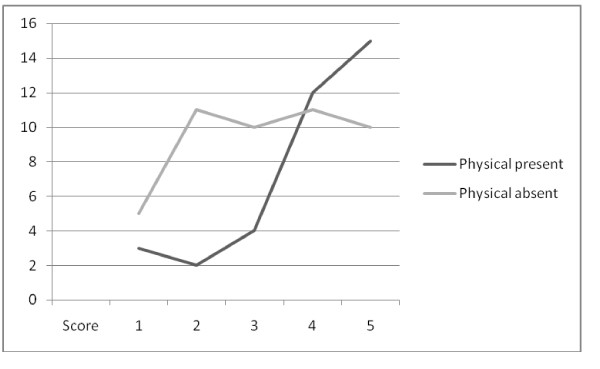
**Follow-up and physical symptoms**. Score of perceived usefulness of follow-up services vs. number of patients describing physical symptoms in the previous month. Patients with symptoms are more likely to perceive follow-up services as beneficial (Mann-Whitney test 2 tailed p < 0.01*(0.0094)).

**Figure 3:**
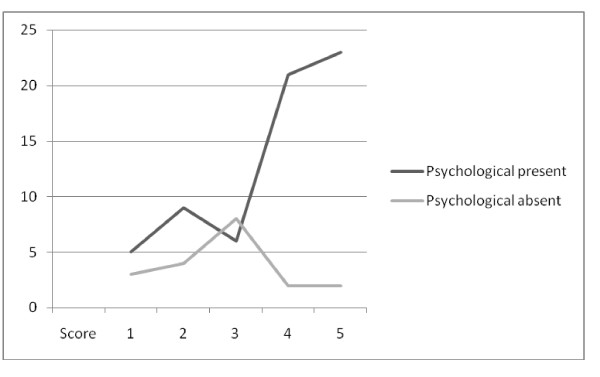
**Follow-up and psychological symptoms**. Score of perceived usefulness of follow-up services vs. number of patients describing psychological symptoms in the previous month. Patients with symptoms are more likely to perceive follow-up services as beneficial (Mann-Whitney test 2-tailed p < 0.01*(0.0036)).

There was a highly statistically significant relationship between the presence of either physical symptoms or psychological symptoms and the desire for follow-up (Figures 2 and 3).

## Discussion

Our study showed that elderly ICU survivors, after ICU discharge, demonstrated a statistically significant difference in the MCS of the SF-36 to younger patients. This positive mental health attitude has been shown by others, but only in sub domains of the SF-36 or specifically in men [8,11,20,21]. This finding of a more positive mental component summary in older patients may have several explanations. It may represent a difference in reason for ICU admission. Studies have shown that HRQOL scores are similar before and after ICU for patients with pre-existing ill-health, while patients suffering sudden and acute pathologies have a marked reduction in score [12,22]. Our results may represent the fact that there are a greater proportion of younger patients in the second category.

A second explanation may be that older patients are better able to cope with the consequences of surviving critical illness than younger people. The similarity in PCS between the two groups would support this. Our study suggests that there may be differing expectations and willingness to accept physical limitations between older and younger patients.

We demonstrated a correlation between hospital length of stay and the physical component summary of the SF 36, the PCS. As Hospital LOS was calculated following discharge from ICU this would represent patients with a prolonged recovery and was expected as physical weakness inevitably impacts on ability to leave hospital. There is also a recognized relationship in ICU survivors between hospital LOS and subsequent development of chronic pain as chronic pain is associated with a poorer HRQOL [20]. Trauma patients, who are often younger, are also known to have prolonged hospital stay on the general ward [23].

We did not demonstrate a correlation between APACHE II scoring and PCS or MCS in ICU survivors. This is consistent with other authors who looked directly at this relationship [5,8].

We assessed other morbidities of interest to us using a series of short questions added to the SF-36 tool as previously advocated [1]. The results demonstrate a large amount of psychological morbidity, a finding consistent with other UK investigators [5,24,25]. The positive correlation between specific ICU-related symptoms and the perceived benefit of a follow-up clinic was marked and although the usefulness of individual symptoms is debatable, that patients with persisting symptoms attributable to ICU stay are more likely to feel follow-up services are beneficial may be useful in screening patients likely to use rehabilitation facilities.

### Strengths and Limitations of the Study

The strength of our study was our attempt to identify a pure general ICU population, using requirement for multiple organ or advanced respiratory support and LOS > 48 hours.

The largest limitation of our study is the response, with a questionnaire return rate of approximately 50%. Although consistent with previous studies using a postal self-administered technique [20,26] it is less than studies using SF 36 administered by other methods [8,11].

The number of patients unable to fully complete the questionnaire was also disappointing providing a response rate for PCS and MCS data of only 37%.

Aware of the potential for selection bias as a result of the low response rate, we looked for differences between the patients responding or not to the questionnaire. The differences in both hospital and ICU readmission rates suggest that the non responders may have still been in hospital or only recently discharged. The fact that the non-responders were younger is not necessarily surprising, but younger and sicker less easy to explain.

The readmission rate to hospital within four months of the ICU survivors was higher than expected [27]. 70% of admissions were related to the primary ICU admission.

In retrospect, the prospective categorization of symptoms into physical and psychological, whilst influenced by SF-36 PCS and MCS, was confusing and open to criticism. If the results had been analysed using simply persisting symptoms vs. perceived benefit of clinic they would have been more statistically significant (Mann-Whitney 2-tailed p < 0.002).

We have used norm based data from a US population to calculate the physical and mental health summary scores. There is limited normative data for the SF-36 on the UK population over 64 and caution has been counseled in its use [28].

Four months may be considered a short survey time for critically ill patients with prolonged ICU stay, the recommendation is 6–12 months [1] and we may have gained more by assessing pre-admission QOL from relatives as previously described [8].

Our study was in a single centre and so may not be generally applicable.

## Conclusion

At 4 months after ICU discharge, the more elderly have significantly better psychological HRQOL as compared with younger patients. Prolonged hospital LOS is associated with a significant reduction in the physical domains of HRQOL. There is a demand for follow-up services and it is related to persisting symptoms at four months identified using a simple questionnaire. The use of a questionnaire to screen patients may identify those likely to attend and needs to be evaluated more fully. Younger patients and those with prolonged hospital lengths of stay have lower quality of life indices and this may have implications for rehabilitation services.

## Abbreviations

ICU: Intensive Care Unit; HRQOL: Health-related quality of life; SF-36: Short Form 36; MCS: Mental component summary of the SF-36; PCS: Physical component summary of the SF-36; LOS: Length of stay.

## Competing interests

The authors declare that they have no competing interests.

## Authors' contributions

FJB designed the study and produced the manuscript. DH assisted in the design of the study and data collection. OFB assisted with study design, performed statistical analysis and on revision of the manuscript. PD assisted with data analysis. All authors read and approved the final manuscript.

## References

[B1] Angus DC, Carlet J (2003). Surviving intensive care: a report from the 2002 Brussels Roundtable. Intensive Care Med.

[B2] Rubenfeld GD, Angus DC, Pinsky MR, Curtis JR, Connors AF, Bernard GR (1999). Outcomes research in critical care: results of the American Thoracic Society Critical Care Assembly Workshop on Outcomes Research. The Members of the Outcomes Research Workshop. Am J Respir Crit Care Med.

[B3] Broomhead LR, Brett SJ (2002). Clinical review: Intensive care follow-up – what has it told us?. Crit Care.

[B4] Jackson JC, Hart RP, Gordon SM, Hopkins RO, Girard TD, Ely EW (2007). Post-traumatic stress disorder and post-traumatic stress symptoms following critical illness in medical intensive care unit patients: assessing the magnitude of the problem. Crit Care.

[B5] Sukantarat K, Greer S, Brett S, Williamson R (2007). Physical and psychological sequelae of critical illness. Br J Health Psychol.

[B6] Dowdy DW, Eid MP, Sedrakyan A, Mendez-Tellez PA, Pronovost PJ, Herridge MS, Needham DM (2005). Quality of life in adult survivors of critical illness: a systematic review of the literature. Intensive Care Med.

[B7] Cheung AM, Tansey CM, Tomlinson G, Diaz-Granados N, Matte A, Barr A, Mehta S, Mazer CD, Guest CB, Stewart TE (2006). Two-year outcomes, health care use, and costs of survivors of acute respiratory distress syndrome. Am J Respir Crit Care Med.

[B8] Cuthbertson BH, Scott J, Strachan M, Kilonzo M, Vale L (2005). Quality of life before and after intensive care. Anaesthesia.

[B9] Graf J, Koch M, Dujardin R, Kersten A, Janssens U (2003). Health-related quality of life before, 1 month after, and 9 months after intensive care in medical cardiovascular and pulmonary patients. Crit Care Med.

[B10] Hofhuis JG, Spronk PE, van Stel HF, Schrijvers GJ, Rommes JH, Bakker J (2008). The impact of critical illness on perceived health-related quality of life during ICU treatment, hospital stay, and after hospital discharge: a long-term follow-up study. Chest.

[B11] Eddleston JM, White P, Guthrie E (2000). Survival, morbidity, and quality of life after discharge from intensive care. Crit Care Med.

[B12] Ridley SA, Chrispin PS, Scotton H, Rogers J, Lloyd D (1997). Changes in quality of life after intensive care: comparison with normal data. Anaesthesia.

[B13] Jones C, Skirrow P, Griffiths RD, Humphris GH, Ingleby S, Eddleston J, Waldmann C, Gager M (2003). Rehabilitation after critical illness: a randomized, controlled trial. Crit Care Med.

[B14] Williams TA, Leslie GD (2008). Beyond the walls: a review of ICU clinics and their impact on patient outcomes after leaving hospital. Aust Crit Care.

[B15] Knaus WA, Draper EA, Wagner DP, Zimmerman JE (1985). APACHE II: a severity of disease classification system. Crit Care Med.

[B16] Ware JE, Kosinski M, Bayliss MS, McHorney CA, Rogers WH, Raczek A (1995). Comparison of methods for the scoring and statistical analysis of SF-36 health profile and summary measures: summary of results from the Medical Outcomes Study. Med Care.

[B17] Chrispin PS, Scotton H, Rogers J, Lloyd D, Ridley SA (1997). Short Form 36 in the intensive care unit: assessment of acceptability, reliability and validity of the questionnaire. Anaesthesia.

[B18] Hofhuis JG, Spronk PE, van Stel HF, Schrijvers AJ, Bakker J (2007). Quality of life before intensive care unit admission is a predictor of survival. Crit Care.

[B19] Ware JE, Koninski M, Dewey JE (2000). How to score version 2 of the SF-36 Health Survey.

[B20] Boyle M, Murgo M, Adamson H, Gill J, Elliott D, Crawford M (2004). The effect of chronic pain on health related quality of life amongst intensive care survivors. Aust Crit Care.

[B21] Abelha FJ, Santos CC, Maia PC, Castro MA, Barros H (2007). Quality of life after stay in surgical intensive care unit. BMC Anesthesiol.

[B22] Wehler M, Geise A, Hadzionerovic D, Aljukic E, Reulbach U, Hahn EG, Strauss R (2003). Health-related quality of life of patients with multiple organ dysfunction: individual changes and comparison with normative population. Crit Care Med.

[B23] Korosec Jagodic H, Jagodic K, Podbregar M (2006). Long-term outcome and quality of life of patients treated in surgical intensive care: a comparison between sepsis and trauma. Crit Care.

[B24] Scragg P, Jones A, Fauvel N (2001). Psychological problems following ICU treatment. Anaesthesia.

[B25] Jones C, Griffiths RD, Humphris G, Skirrow PM (2001). Memory, delusions, and the development of acute posttraumatic stress disorder-related symptoms after intensive care. Crit Care Med.

[B26] Deja M, Denke C, Weber-Carstens S, Schroder J, Pille CE, Hokema F, Falke KJ, Kaisers U (2006). Social support during intensive care unit stay might improve mental impairment and consequently health-related quality of life in survivors of severe acute respiratory distress syndrome. Crit Care.

[B27] Keenan SP, Dodek P, Chan K, Simon M, Hogg RS, Anis AH, Spinelli JJ, Tilley J, Norena M, Wong H (2004). Intensive care unit survivors have fewer hospital readmissions and readmission days than other hospitalized patients in British Columbia. Crit Care Med.

[B28] Brazier JE, Harper R, Jones NM, O'Cathain A, Thomas KJ, Usherwood T, Westlake L (1992). Validating the SF-36 health survey questionnaire: new outcome measure for primary care. BMJ.

